# Psychometric Properties of the Wong and Law Emotional Intelligence Scale in a Colombian Manager Sample

**DOI:** 10.3390/jintelligence10020029

**Published:** 2022-05-15

**Authors:** Julio César Acosta-Prado, Rodrigo Arturo Zárate-Torres, Arnold Alejandro Tafur-Mendoza

**Affiliations:** 1 School of Business Science, Universidad del Pacífico, Lima 15072, Peru; 2 School of Management, Universidad Externado de Colombia, Bogota 111711, Colombia; 3 Colegio de Estudios Superiores de Administración, Bogota 111071, Colombia; rodrigo.zarate@cesa.edu.co; 4 Research Center (CIUP), Universidad del Pacífico, Lima 15072, Peru; pcpsataf@upc.edu.pe

**Keywords:** WLEIS, emotional intelligence, managers, psychometric properties, reliability, validity

## Abstract

Within the organizational field, emotional intelligence is linked to socially competent behaviors, which allow the development of labor and organizational abilities necessary for professional development. Thus, in workers, emotional intelligence is related to a wide range of organizational variables. The purpose of the present study was to evaluate the psychometric properties of the Wong and Law Emotional Intelligence Scale (WLEIS) in the Colombian context, specifically, in a population of managers. The study was instrumental. The sample consists of 489 Colombian managers, obtained through non-probability sampling (a purposive sample), who work in companies located in Bogota. The results indicated that the four-factor oblique model presents favorable fit indices, as well as the higher-order model, the latter having additional theoretical support. These results indicate that it is possible to consider partial scores for each of the four factors of the WLEIS, as well as an overall emotional-intelligence score. Also, the WLEIS scores have validity evidence based on relations to other variables (convergent and discriminant evidence) and are reliable. These first findings for Colombian managers contribute to the accumulation of international evidence of emotional intelligence measured with the WLEIS.

## 1. Introduction

Emotional intelligence (EI) is a social ability that allows a person to control his or her own emotions and understand the emotions of others, which is essential for any professional today, from general staff to those in management positions (Mayer et al. 2016; Salovey and Mayer 1990). In this sense, EI can be understood as a set of interrelated abilities that people possess to function in society, combining intrapersonal and interpersonal abilities (Law et al. 2004). The study of EI began in the last decade of the last century and since then a large body of knowledge on the subject has been formed in various fields of social and behavioral sciences, for example, the labor or organizational field regarding how people cope with adverse or stressful events (Boyatzis 2018). Therefore, EI allows people to better adapt to the demands of their environment in a dynamic context, such as the one in which companies are currently immersed (Kotsou et al. 2018).

EI has been studied in the workplace as a predictor of performance, organizational commitment, and leadership (Jain and Duggal 2018). Precisely, the relationship between EI and leadership has been investigated more frequently in managers or decision makers within companies, finding that people with better leadership styles (for example, transformational), have higher levels of EI, which allows them to obtain better results in their workplaces (Miao et al. 2018; Issah 2018). Likewise, there is still little evidence on the influence that low levels of EI have on problematic behaviors such as communication difficulties or poor collaborative-work abilities (Kircaburun et al. 2019; Pérez-Fuentes et al. 2019). Therefore, when working with the EI construct, it is not enough to analyze it globally as a unidimensional variable; it is necessary to evaluate it considering the different abilities that compose it, that is, from a multidimensional perspective.

Regarding the theoretical models developed for the study of EI, they can be grouped into three main categories: EI as (1) a trait, (2) an ability, or (3) mixed, which involves a combination of the two previous ones (O’Connor et al. 2019). This proposed classification derives from the way EI is measured. As a trait, EI presents an overlap with personality traits (e.g., emotional regulation or empathy) and is assessed by self-report instruments (Salovey and Mayer 1990). On the other hand, EI as an ability involves the processing of information from the self and others and can therefore be assessed using peak performance tests (Mayer and Salovey 1997). However, self-reports can also be used to measure EI as an ability, among which the WLEIS stands out, due to its stable structure and internal consistency (Bru-Luna et al. 2021).

Although there is some controversy as to whether the WLEIS is a measure of EI as an ability, because some studies identify this scale as a measure of EI as a trait (Pérez et al. 2005; Brannick et al. 2009), further research is still needed regarding the measurement of EI using the WLEIS, as other studies provide support that this test measures EI as an ability (LaPalme et al. 2016; Law et al. 2004). Finally, mixed EI refers to instruments that measure a combination of traits, skills and competencies (Bar-On 1997).

In Colombia, several studies have been carried out on EI and its relationship with various organizational variables in workers and managers to propose programs to improve the EI of the people involved (Acosta-Prado et al. 2015; Acosta-Prado and Zárate 2017). For the measurement of EI, self-report measures are mostly used, among which are the Emotional Quotient Inventory (EQ-i), Trait Meta-Mood Scale (TMMS), Trait Emotional Intelligence Questionnaire (TEIQue), and Wong and Law’s Emotional Intelligence Scale (WLEIS) (Bru-Luna et al. 2021). The latter instrument has been used in samples of company managers from different economic sectors (Acosta-Prado et al. 2015). However, there are no previous studies that exhaustively examine its psychometric properties to justify its use with the aforementioned sample and have theoretical and empirical support for the correct interpretation of the scores.

The WLEIS is a self-report measure developed by Wong and Law (2002), consisting of 16 items to measure EI based on the revised model of Mayer and Salovey (Salovey and Mayer 1990; Mayer and Salovey 1997). The instrument is composed of four dimensions: (1) self-emotion appraisal, (2) others’ emotion appraisal, (3) use of emotion, and (4) regulation of emotion (Wong and Law 2002). Self-emotion appraisal refers to people’s awareness of their feelings and thoughts about those feelings. Others’ emotion appraisal is linked to the perception and understanding of other people’s emotions. The use of emotion involves monitoring, evaluation, and control measures to modify one’s feelings. Regulation of emotion enables people to improve their performance through self-motivated emotions.

The WLEIS was proposed for leadership and management studies, although it was gradually extended to various organizational, educational, and clinical areas, among others (Keefer et al. 2018; Law et al. 2004). In the original study of the WLEIS, the authors tested various models to determine the structural composition of the instrument, among them, the model of four related factors and another of a higher order (four first-order factors and one second-order factor). The latter model was the one with the greatest empirical support that was in line with the theory on which the instrument was developed (Wong and Law 2002). However, psychometric studies carried out in various countries show the existence of four factors, although the general factor is questionable.

In a sample of South Korean nurses, the WLEIS presented a four-factor related factor structure, good reliability levels (α between 0.88 and 0.92 at the factor level and 0.91 for the total scale), adequate levels of item discrimination (r_itc_ > 0.40), and the absence of floor and ceiling effects (Park and Yu 2021). In China, the WLEIS was validated in a sample of university students, using a version with five response options in a Likert format, which showed good fit indices for the four related factors model, the higher-order model, and the bifactor model; the latter was slightly better than the previous ones, while the reliability for the four specific factors and the general factor was good (α > 0.70) (Di et al. 2021). In the same country, in a sample of the general population, it was found that the factor model with the best fit was the four related factors (Kong 2017). In Morocco, the adaptation of the WLEIS to university students provided support for a higher-order structure (four first-order factors and one second-order factor) (Ghoudani et al. 2018). In Nepal, using a five-point Likert scale version of the WLEIS, the related four-factor structure, as well as the invariance of the model, was corroborated with a sample of UK university students (Sochos et al. 2021).

In Italy, in a sample of the general population, good reliability levels were found for the factors and the total test (α > 0.80), as well as the adequacy of the data to an oblique factorial model (four related factors) and a higher-order model (four lower-order factors and one higher-order factor) (Iliceto and Fino 2017). In Spain, the adaptation distributed among the general population indicated a good reliability level at the total level (α > 0.91) and in the dimensions (α between 0.79 and 0.84), the moderate relationship between EI and subjective happiness (r = 0.38), and a factorial structure of four related factors, with factor loadings between 0.57 and 0.85 (Extremera Pacheco et al. 2019). On the other hand, in a sample of medical students from Spain and Portugal, it was found that the factor structure that best fit the WLEIS was the model of four related factors, which evidenced good reliability levels (α > 0.80) and an adequate discriminative ability of the items (Carvalho et al. 2016).

At the Latin American level, in Chile, the WLEIS was studied in a sample of managers, a structural model of four related factors was found to have the best fit, although with low reliability levels in the factors (α < 0.70), except the factor regulation of emotion (α = 0.82) (Acosta-Prado and Zárate 2019). In Peru, the internal structure of the WLEIS was studied in adults, a structure of four related factors was found to have the best fit, high factor loadings of the items and moderate and large correlations between factors, as well as a good reliability level for the factors (α > 0.70) (Merino et al. 2016). Likewise, in nursing students, the same results were corroborated, with a good discriminative ability of the items evaluated through item-rest correlation also being reported (Merino-Soto et al. 2019).

These studies corroborate the presence of the four factors in the WLEIS, although only in some of them is the general factor present, either underlying the items (a bifactor model) or the specific factors (a higher-order model). Therefore, when conducting psychometric studies, it is necessary to test these models to find the one that best fits the data. In this way, we will have a better approximation for the measurement of EI, a starting point for the planning of actions that allow its training and improvement and providing the necessary tools to people for their adequate development in society and personal wellbeing (Malinauskas and Malinauskiene 2020).

However, the psychometric properties of instruments that measure EI in managers in Colombia have not been evaluated. Therefore, the present study aims to analyze the psychometric properties of the WLEIS in a sample of Colombian managers. This involves providing reliability evidence to the test scores through the internal-consistency method, as well as validity evidence from two sources, internal structure, and relations to other variables (convergent and discriminant evidence). For this second validity evidence, a measure of subjective happiness was additionally used, because, theoretically and empirically, this variable and EI present a significant relationship (Extremera Pacheco et al. 2019; Blasco-Belled et al. 2020; Ghahramani et al. 2019). Thus, the Subjective Happiness Scale (SHS) was used. Instrumental studies of the SHS in Latin America report adequate psychometric properties in countries such as Chile (Vera-Villarroel et al. 2011), Argentina (Ortiz et al. 2013), and Puerto Rico (González-Rivera 2021).

## 2. Materials and Methods

### 2.1. Design

The present study followed an instrumental design because the psychometric properties of an instrument that measures EI were analyzed (Ato et al. 2013). In this sense, evidence of validity and reliability was provided to justify the use of the instrument in the organizational field within the Colombian context. In the methodological process of the study, the recommendations proposed by the standards for educational and psychological testing (American Educational Research Association 2014) were followed, as well as the good practices in the development and psychometric evaluation of tests in organizational research (Zickar 2020).

### 2.2. Participants

Participants were collected through a non-probabilistic sampling, purposive type, that sought to maintain a balance between the different categories of sociodemographic variables (Kerlinger and Lee 2000). Regarding the number of participants, the required sample size for a study using structural equation model (SEM) was calculated through the online calculator developed by Soper (2022), considering the model used for the relationship between EI and happiness in the collection of validity evidence based on relations to other variables. The parameter values used for the calculation of the required sample size were expected effect size of 0.30, a desired statistical power level of 0.95, the number of latent variables was 5 (4 EI factors and 1 happiness factor), the number of observable variables was 20 (16 EI and 4 happiness) and a probability level (α) of 0.05. The results indicated a recommended minimum sample size of 223.

To achieve greater robustness and stability of the statistical analyses performed, a larger sample size than the minimum recommended was collected. Accordingly, the WLEIS was applied to 498 people. In addition, for the study to collect validity evidence based on relations to other variables, the SHS was applied to 167 people. However, after the database cleaning described in the procedure section, the final sample was composed of 489 participants who answered the WLEIS and 151 participants who answered both the WLEIS and the SHS. The participants were Colombian managers working in companies located in Bogota city. A description of the main characteristics of the study sample is presented in Table 1, which highlights that most of the participants belonged to companies in the ICT sector (n = 300, 62.24%) and had 1 to 3 years of experience (n = 164, 33.88%).

### 2.3. Measures

#### 2.3.1. Wong and Law Emotional Intelligence Scale (WLEIS)

The Wong and Law Emotional Intelligence Scale is a self-report scale designed by Wong and Law (2002) to measure EI based on 16 items grouped into four factors (four items per factor): self-emotion appraisal (SEA), others’ emotion appraisal (OEA), use of emotion (UOE), and regulation of emotion (ROE). The WLEIS response format includes seven-point Likert-type questions, ranging from 1 (strongly disagree) to 7 (strongly agree). Higher scores indicate a higher level of EI. For this study, the Spanish version of the WLEIS (WLEIS-S) was used, which replicates the original four-factor structure and for which the reliability of the scores was adequate both in the factors and at the global level (Extremera Pacheco et al. 2019). Likewise, the WLEIS has shown good psychometric properties in samples of managers in several Latin American countries (Acosta-Prado et al. 2015; Acosta-Prado and Zárate 2017, 2019).

#### 2.3.2. Subjective Happiness Scale (SHS)

This instrument was developed by Lyubomirsky and Lepper (1999) to assess global subjective happiness. For this study, the version adapted to Spanish by Quezada et al. (2016) for a Mexican sample was used. The SHS is composed of four items with a unidimensional structure underlying them. The response format of the four items is a seven-point Likert-type. The total score on the SHS is obtained from the average of the responses to the four items (recoding item 4).

The psychometric properties of the SHS for the present study were good. Regarding validity, evidence based on the internal structure from the CFA showed an adequate fit in favor of the unidimensionality of the scale (χ^2^ = 2.276, df = 2, χ^2^/df = 1.138, CFI = 1.000, TLI = 0.999, RMSEA = 0.030 [90% CI 0.000; 0.168], SRMR = 0.017, and WRMR = 0.191), as well as high factor loadings ranging from 0.561 to 0.873. In addition, validity evidence based on relations to other variables evaluated through the average variance extracted (AVE) presented good results in favor of convergent evidence with a value of 0.594, higher than 0.50, the threshold used for the AVE. Finally, regarding reliability, the categorical omega coefficient (ω_u-cat_) obtained a point estimate of 0.763 with a 95% bias-corrected and accelerated (bca) bootstrap confidence interval at 10,000 replications between 0.675 and 0.818. Therefore, the scores show an adequate reliability level.

### 2.4. Procedure

Data were collected between August 2019 and March 2020. The instruments were delivered in person in different meetings with the participants. All participants expressed their authorization to use the data provided. Also, the researchers stated the ethical consideration that the answers are completely anonymous and that all the data collected will be ethically treated. Participants could continue completing the questionnaires only if they provided consent. All the results were anonymized by encrypting the data. It was not possible for the researchers to identify any of the research participants. Every effort was taken to protect the privacy and maintain confidentiality of the information acquired from the participation in the study.

Initially, 498 responses were collected for the WLEIS, wherein eight protocols, a maximum of two items, were observed with missing values (0.10%); so, we proceeded with the imputation of these values using the logistic regression technique (Kang 2013). Subsequently, a univariate outlier analysis was performed (Leys et al. 2019), finding nine cases, which were removed from the database, leaving the final sample with 489 participants. Regarding the responses to the SHS: there were initially 167 cases, and two missing values were detected, which were imputed through logistic regression. After completing the database, the outlier analysis was performed, three univariate outliers (Leys et al. 2019) and 13 multivariate outliers (Leys et al. 2018) were found and 16 cases were discarded from the database, leaving a final sample of 151 participants for the study of validity evidence based on relations to other variables.

### 2.5. Data Analysis

The descriptive statistics of the items were obtained through the mean, standard deviation, skewness, and kurtosis. These last two coefficients indicated the level of departure from a normal distribution, considering adequate values between -2 and 2 (Tabachnick and Fidell 2019). Likewise, the floor effect and ceiling effect of the items were analyzed, considering the percentage of people who answered the lowest and highest answer alter-native, respectively. The items with percentages equal to or less than 15% were evaluated as free of these effects (McHorney and Tarlov 1995). Additionally, the discrimination of the items was estimated through the item–rest polyserial correlation, both at the level of dimensions and the total level, taking as acceptable indices greater than 0.20 (Schmeiser and Welch 2006).

Validity evidence was collected based on the internal structure of the test using confirmatory factor analysis (CFA). The estimation method was the weighted least squares means and variance adjusted (WLSMV) with robust standard errors and scaling-shifted scaled statistic test (SS), applied to the matrix of polychoric correlations of the items. Regarding the goodness-of-fit indices to assess the estimated models, the ratio between chi-square and degrees of freedom (SSχ^2^/df), taking as appropriate values below 5 (Schumacker and Lomax 2016); the comparative fit index (CFI) and Tucker–Lewis index (TLI), with adequate values higher than 0.90 and good values higher than 0.95 (Keith 2019); the root mean square error of approximation (RMSEA) and standardized root mean square residual (SRMR), considering values less than 0.08 as adequate and good values less than 0.06 (Schumacker and Lomax 2016); and the weighted root mean square residual (WRMR), with values lower than 1.00 being appropriate (DiStefano et al. 2018), were used. To compare models with a good fit, the differences (Δ) in CFI, TLI, and RMSEA were used, considering models significantly different when ΔCFI > 0.010, ΔTLI > 0.010, and ΔRMSEA > 0.010 (Rutkowski and Svetina 2017). For the interpretation of the bifactor model, the general hierarchical omega coefficient (ω_h_) and of the subscales (ω_hs_) were considered as additional indices, with values greater than 0.30 for the latter being taken as substantial (Rodriguez et al. 2016). The explained common variance (ECV) and the percentage of uncontaminated correlation (PUC) were used, where values greater than 0.70 in both supports unidimensionality (Dominguez-Lara and Rodriguez 2017). The item-level explained common variance (IECV) was used, where values above 0.80 are expected (Rodriguez et al. 2016).

Validity evidence was collected based on relations to other variables. For this purpose, convergent and discriminant evidence was collected. The convergent evidence was evaluated from the average variance extracted (AVE), taking as minimum acceptable values those proposed by Moral (2019), which considers the factor loadings, the reliability coefficient, and the number of factor items evaluated. A structural equation model (SEM) was tested to estimate the relationship between EI and happiness, using the same criteria as in the CFA to assess model fit. In addition, the relationship between the variables was assessed as small, medium and large considering correlation coefficients above 0.10, 0.30 and 0.50, respectively (Cohen 1988). On the other hand, the discriminant evidence was collected through two procedures, the heterotrait–monotrait ratio (HTMT2), considering the generic measurement model (Roemer et al. 2021), taking as adequate values lower than 0.85 (Henseler et al. 2015), and the Fornell and Larcker criterion, which consists of comparing the square root of the AVE and the correlations with the other variables, where the former must be greater than the latter to conclude that there is discriminant evidence (Fornell and Larcker 1981).

The reliability of the test scores was evaluated using the internal-consistency method. The categorical omega coefficient (ω_cat_) was used, estimated from the factorial solution obtained from the confirmatory factor analysis (Flora 2020; Viladrich et al. 2017), and valued as adequate from 0.70 (Nunnally and Bernstein 1994). Additionally, to obtain a better understanding of the score reliability, inter-item polychoric correlations were estimated (Ventura-León and Peña-Calero 2021).

The data analysis was carried out on R version 4.1.3 (R Core Team 2022) in RStudio (RStudio Team 2022). The tidyverse package version 1.3.1 (Wickham et al. 2019) was used for data manipulation; the Routliers package version 0.0.0.3 (Klein and Delacre 2021) for the identification of outliers; the psych package version 2.2.3 (Revelle 2022) for item analysis; the lavaan package version 0.6–10 (Rosseel 2012) for the CFA; the semTools package version 0.5–5 (Jorgensen et al. 2021) to estimate the reliability and AVE; the CTT package version 2.3.3 (Willse 2018) to estimate the polyserial item-rest correlation; the MBESS package version 4.9.0 (Kelley 2022) for the estimation of confidence intervals for reliability coefficients; the naniar package version 0.6.1 (Tierney et al. 2021) to summarize and visualize missing data; the TestDataImputation package version 2.3 (Dai et al. 2021) for missing item responses imputation; and the BifactorIndicesCalculator package version 0.2.2 (Dueber 2021) for the calculation of estimators complementary to the bifactor model. Finally, an online tool was used to estimate HTMT2 (Henseler 2022).

## 3. Results

### 3.1. Item Analysis

Table 2 shows the descriptive analysis of the WLEIS items. The central tendency of the items indicated that participants opted to choose the highest response options, as the means were between 5.00 (ROE_3) and 6.06 (UOE_4). In addition, the dispersion of responses was low where the standard deviation of the items was close to 1, ranging from 1.03 (UOE_4) and 1.47 (ROE_3). Regarding the shape measures, the skewness and kurtosis coefficients of the 16 WLEIS items were found to be within the range of −2 and 2, indicating that the distributions of the responses do not substantially deviate from a normal distribution. Regarding the analysis of the response options, ceiling effects were observed for most of the items: all items of the SEA and UOE factors, as well as items OEA_2 and OEA_3 of the OEA factor. Likewise, although no floor effects were found, in items SEA_2 and UOE_4 no responses were found in the lowest alternative (strongly disagree). Finally, all the items showed a good discriminative capacity, both at the global level and for each factor, since the item–rest polyserial correlation coefficients were greater than 0.20, with values from 0.261 (OEA_3 at the global level) to 0.872 (ROE_4 at the factorial level).

### 3.2. Validity Evidence Based on the Internal Structure

Table 3 presents the results of the models tested through the CFA. Five models were tested for the factor structure of the WLEIS, in order to determine the presence of four specific factors and one general factor, although with different specificities in each model. The unifactorial model, which implied the presence of a single general factor (EI) underlying the 14 items, was the one that presented the worst fit indices, followed by the orthogonal model, which, although it posited the presence of four specific factors, assumed no relationship between them (χ^2^/df > 5; RMSEA > 0.08; CFI < 0.90; TLI < 0.90; SRMR > 0.08; and WRMR > 1.00).

On the other hand, a bifactor model was tested, where an orthogonal model and a unifactorial model were evaluated simultaneously. The bifactor model presented good fit indices (χ^2^/df < 5; RMSEA < 0.08; CFI > 0.95; TLI > 0.95; SRMR < 0.06; and WRMR < 1.00). However, because bifactor models usually tend to show adequate fit indices, additional statistics were calculated for interpretation. In this regard, the general factor only explained 37.19% (ω_h_ = 0.670) of the variability in the scores, which is not large enough to consider it independently. Conversely, the specific factors provided substantial information (ω_hs_ > 0.30; OEA = 0.622, UOE = 0.596, and ROE = 0.639), except for SEA (ω_h_ = 0.097). In relation to the amount of explained common variance, this is below the expected (ECVtotal = 0.357; PUC = 0.800). Likewise, the items were more influenced by the specific factors than by the general factor (IECV_mean_ = 0.377; IECV_median_ = 0.294). Considering these results, it is concluded that the bifactor model shows a low influence of the general factor on the items, compared to the specific factors.

**Figure 1:**
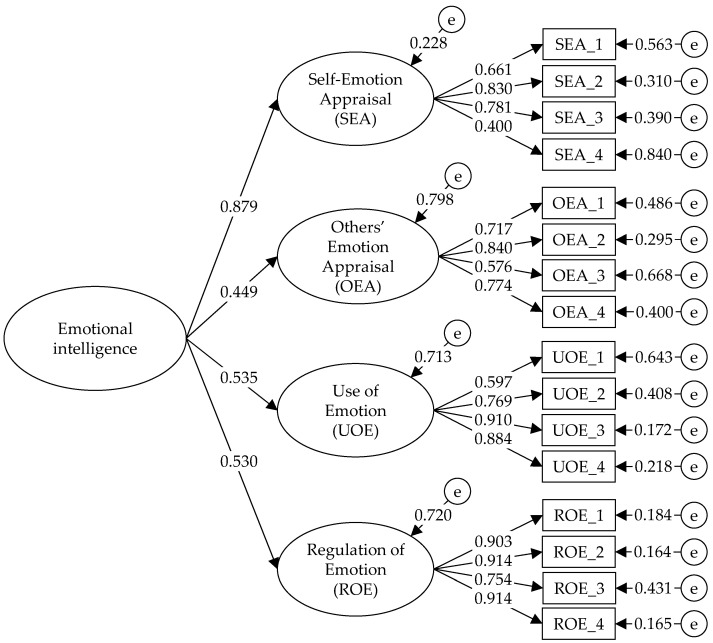
Factorial structure of the higher-order model of the WLEIS.

Finally, an oblique model of four related factors and a higher-order model with a second-order factor underlying four first-order factors were tested. In both models, the fit indices were good (χ^2^/df < 5; RMSEA < 0.08; CFI > 0.95; TLI > 0.95; and SRMR < 0.06), with no significant differences between them (ΔCFI < 0.010, ΔCFI < 0.010 and ΔRMSEA < 0.010). In the oblique model, the items presented high factor loadings (between 0.401 and 0.914), as well as moderate correlations (greater than 0.40) between the SEA factor and the other factors, denoting the possible presence of a higher factor. In the higher-order model, the factor loadings of the items were high (between 0.400 and 0.914) and the factor loadings of the first-order factors in the second-order factor were also high (above 0.40). Thus, due to the empirical evidence and based on the theoretical model of Mayer and Salovey (1997), the higher-order model is the one that best conceptualizes EI measured through the WLEIS (Figure 1).

### 3.3. Reliability

Table 4 presents the results of the reliability estimation using the categorical omega coefficient of the WLEIS higher-order model. The responses of the first-order factors showed adequate reliability levels (ω_h-ss-cat_ > 0.70), with values between 0.755 (SEA) and 0.914 (ROE). Likewise, in the second-order factor, the reliability of the responses was slightly below 0.70 (ω_h-ss-cat_ = 0.628). Additionally, the mean inter-item polychoric correlations (r_i-i_) showed values greater than 0.30 for the first-order factors and slightly below this value for the second-order factor (mean r_i-i_ = 0.273). The full inter-item polychoric correlations are reported in Table 5. Thus, the internal consistency analysis of the items provides evidence of reliability for the four lower-order factors, while the reliability of the higher-order factor is questionable.

### 3.4. Validity Evidence Based on Relations to Other Variables

Table 4 presents the results of the convergent and discriminant validity evidence. Regarding convergent evidence, the higher-order model of the relationship between EI and subjective happiness showed good fit indices (χ^2^ = 260. 977, df = 165, χ^2^/df = 1.582, CFI = 0.971, TLI = 0.966, RMSEA = 0.062 [90% CI 0.048; 0.076], SRMR = 0.081, and WRMR = 0.923), with a large positive correlation of 0.552 between the two variables. Likewise, the oblique model of the relationship between the four factors of EI and subjective happiness also presented adequate fit indices (χ^2^ = 230.436, df = 160, χ^2^/df = 1.440, CFI = 0.979, TLI = 0.975, RMSEA = 0.054 [90% CI 0. 038; 0.069], SRMR = 0.068, and WRMR = 0.778), where the correlations between the variables were positive and moderate (greater than 0.30), except for the relationship between others’ emotion appraisal and subjective happiness, which was null or trivial (less than 0.10). On the other hand, the AVE for the others’ emotion appraisal, use of emotion, and regulation of emotion factors was greater than 0.50, whereas, for the factor self-emotion appraisal, the AVE was slightly below 0.50. However, because this factor presented high factor loadings and a reliability coefficient greater than 0.70, an AVE greater than 0.37 can be considered acceptable for the four items (Moral 2019).

Regarding the discriminant evidence, the HTMT2 was less than 0.85 in all variables, with a range between 0.193 and 0.592, which is acceptable in all cases. These results were supported by the Fornell–Larcker criterion, since the square root of the AVE of the factors of EI and subjective happiness was higher than the correlations between the same variables. Therefore, considering the results obtained, it is possible to conclude that the WLEIS scores have validity evidence based on relations to other variables (convergent and discriminant evidence).

## 4. Discussion

This study aimed to analyze the psychometric properties of the WLEIS in a sample of Colombian managers. For this purpose, reliability and validity evidence was collected from different statistical procedures. Regarding validity, the evidence based on the internal structure using the CFA indicated that the model with the greatest theoretical and empirical support was the higher-order model. In this model, four first-order factors (self-emotion appraisal, others’ emotion appraisal, use of emotion, and regulation of emotion) explained the 16 items that make up the WLEIS, while a second-order factor (EI) explained all four factors. Regarding the evidence based on relations to other variables, EI, and its factors, had moderate and high correlations with subjective happiness, except the factor others’ emotion appraisal. Another method used for convergent evidence was the AVE, which showed acceptable values for the WLEIS factors. For the discriminant evidence, two statistical methods were also used, the HTMT2 and the Fornell–Larcker criterion, which provided support for the factors of the instrument. Finally, the reliability of the WLEIS scores was estimated through the internal-consistency method, presenting good levels for the first-order factors and a low level for the second-order factor.

Item discrimination analyzed using the item–rest polyserial correlation, showed that all items had a good discriminative capacity, that is, they differentiated between people with low and high scores on the test, considering all items as a whole, as well as each group of items in their corresponding factor. These results coincide with those found in South Korea in a sample of nurses, where the item–rest correlations were greater than 0.40 (Park and Yu 2021), and with those reported in Peru, in a sample of nursing students (Carvalho et al. 2016). On the other hand, in the analysis of the response options, it was observed that few people marked the lowest alternatives: even in the SEA_2 and UOE_4 items, no person selected the option “strongly disagree”. In addition, a ceiling effect was observed for most of the WLEIS items, where a proportion greater than 15% selected the “strongly agree” alternative. This is probably due to a social desirability effect on the part of the participants and the characteristics they have as business leaders, so it is necessary to analyze strategies to avoid the influence of bias in the responses.

Regarding the factor structure of the WLEIS, the model with the best-fit indices was the higher-order model, which has the theoretical support of the revised model of Mayer and Salovey (Salovey and Mayer 1990; Mayer and Salovey 1997). These results coincide with what was found in China (Di et al. 2021), Morocco (Ghoudani et al. 2018), Italy (Iliceto and Fino 2017), and the initial study by Wong and Law (2002), which also reported four first-order factors and one second-order factor. Although the related four-factor model showed a good fit and was not significantly different from the higher-order model, the theoretical aspect was prioritized. In this sense, EI is a set of interrelated abilities that allows the adequate development of a person in society (Mayer and Salovey 1993). Therefore, underlying these abilities, there is a global latent variable that generates specific behaviors, which we call EI. Finally, although the bifactor model allows a simultaneous evaluation of a general factor and specific factors, it did not have sufficient empirical support in this study, unlike that reported in China (Di et al. 2021).

Regarding the relations to other variables, for convergent evidence, WLEIS scores were related to a measure of subjective happiness. The correlation between EI and subjective happiness was 0.552, implying a strong relationship between the variables. These results agree with the work of Blasco-Belled et al. (2020), Ghahramani et al. (2019), and Extremera Pacheco et al. (2019), who reported correlation coefficients of 0.45, 0.46, and 0.44, respectively. At the factor level, the correlations were moderate (greater than 0.30), except others’ emotion appraisal, similar to those reported by Extremera Pacheco et al. (2019). The AVE for each of the factors also showed adequate results, so it is possible to conclude that the WLEIS has convergent evidence. Regarding the discriminant evidence, the HTMT2 and the Fornell–Larcker criterion reported favorable results for the WLEIS. Finally, the reliability evidence indicated adequate levels of internal consistency for the four first-order factors, similar to that reported in previous studies (Merino et al. 2016; Extremera Pacheco et al. 2019; Merino-Soto et al. 2019; Blasco-Belled et al. 2020). However, the second-order factor showed a value below 0.70, so its interpretation should be performed considering this result.

The theoretical contribution of the study lies in providing empirical support to the revised model of Mayer and Salovey (Salovey and Mayer 1990; Mayer and Salovey 1997), which proposes the presence of specific abilities and a global component called EI. In this way, this study joins other research developed in different countries that provides evidence of the transculturality of the EI model used. In this way, the findings are linked to the theory developed in the introduction. Regarding the practical contribution of the study, it provides an instrument with adequate psychometric properties that justifies its use in the organizational field, specifically in Colombian managers, so that, due to the characteristics of the instrument, it can be useful in different processes or stages of a company. This allows the WLEIS to be used in the workplace, just as other research justifies its use in other fields such as education and sports, among others. Likewise, the WLEIS can contribute as a measurement tool in intervention programs to evaluate their effectiveness or to establish a baseline to help work with people who have greater difficulties in managing their emotions.

Finally, the limitations of the study consist of the characteristics of the sample used (the majority of participants had completed university studies and worked in the ICT sector), since it is a specific group within the companies, although of special interest in these issues related to management and leadership. In this way, the generalization of the results is limited to other groups with similar characteristics. Likewise, for the analysis of the relationship with other variables, the sample size used (151 participants) was smaller than the minimum recommended because fewer people responded to both instruments for personal reasons that were beyond the researchers’ control. Therefore, the interpretation of this specific analysis should be performed considering this limitation and making a cautious conclusion. Therefore, it is necessary to replicate this finding in a larger sample to reaffirm the findings of the present study.

Also, since the WLEIS was applied to managers and the presence of falsified answers is possible, it is necessary to carry out studies of biases in the answers obtained in the test, to avoid distortions in the results, for example, the study of neglected answers (Merino-Soto et al. 2021). Future studies should also consider analyzing the psychometric properties of the WLEIS employing the Rasch measurement theory (RMT) or item response theory (IRT), since they allow a more-detailed analysis of the response options. This is because, in this study, a low rate of people chose the lowest options and, on the contrary, a high rate of marking the highest alternatives was observed. Therefore, it may be necessary to collapse some response options to improve the quality of the instrument.

## 5. Conclusions

The present study demonstrates that the WLEIS has adequate psychometric proper-ties in Colombian managers of companies located in Bogota belonging to different eco-nomic sectors, although mainly the ICT sector. Considering the internal structure of a higher order was found, the WLEIS allows obtaining scores in the four first-order factors that it evaluates, as well as a total score of EI. This makes it possible to use the WLEIS as a broad measure of EI or to identify more specific aspects of this variable, at least in the four factors that this scale evaluates. Also, given its small number of items, it is efficient when used in selection processes, program-participant assignment, mental-health evaluations at work, and research projects involving the studied population. Although additional validity evidence is required, since validation implies a continuous process of gathering empirical evidence under the theory, these first findings for Colombian managers contribute to the accumulation of international evidence on the measurement of EI through the WLEIS.

## Figures and Tables

**Table 1:** Sociodemographic characteristics of participants.

Variable	Category	n	%
Age	20 years or less	1	0.21
	21 to 25 years	42	8.68
	26 to 35 years	255	52.69
	36 to 45 years	133	27.48
	46 to 60 years	51	10.54
	61 years or more	2	0.41
Sex	Female	247	51.03
	Male	237	48.97
Educational level	High school complete	3	0.62
	University incomplete	16	3.31
	University complete	232	48.03
	Postgraduate	232	48.03
Time in current position	2 years or less	178	36.93
	2 to 5 years	179	37.14
	5 to 10 years	81	16.80
	10 years or more	44	9.13
Employees under charge	No employees	174	36.10
	1 to 2 employees	81	16.80
	3 to 10 employees	125	25.93
	11 to 20 employees	37	7.68
	20 employees to more	65	13.49
years of work experience	No work experience	101	20.87
	1 to 3 years	164	33.88
	4 to 7 years	111	22.93
	8 years or more	108	22.31
Economic sector	Trade	31	6.43
	Communications	33	6.85
	Construction	16	3.32
	Finance	64	13.28
	Industrial	33	6.85
	ICT	300	62.24
	Transportation	5	1.04

**Table 2:** Item analysis and discrimination.

Item	Mean	Standard Deviation	Skew	Kurtosis	Item–Rest (Global)	Item–Rest (Factor)	Floor (%)	Ceiling (%)
SEA_1	5.60	1.18	−1.02	1.07	0.461	0.522	0.20	22.29
SEA_2	5.62	1.08	−0.88	0.81	0.581	0.632	0.00	20.25
SEA_3	5.56	1.13	−0.86	0.77	0.552	0.635	0.20	19.84
SEA_4	5.56	1.33	−0.90	0.38	0.292	0.221	0.61	27.81
OEA_1	5.23	1.13	−0.57	0.25	0.366	0.548	0.20	11.45
OEA_2	5.49	1.20	−0.89	0.69	0.406	0.707	0.41	19.84
OEA_3	5.49	1.27	−0.91	0.59	0.261	0.510	0.61	22.29
OEA_4	5.36	1.06	−0.62	0.65	0.464	0.681	0.20	12.47
UOE_1	5.92	1.06	−1.04	1.09	0.376	0.558	0.20	34.97
UOE_2	5.92	1.18	−1.41	1.93	0.432	0.745	0.20	36.20
UOE_3	5.92	1.08	−1.26	1.94	0.533	0.825	0.20	33.33
UOE_4	6.06	1.03	−1.27	1.71	0.527	0.809	0.00	40.08
ROE_1	5.13	1.30	−0.67	0.16	0.589	0.828	0.82	13.29
ROE_2	5.18	1.24	−0.65	0.13	0.586	0.844	0.20	12.47
ROE_3	5.00	1.47	−0.67	−0.15	0.493	0.704	1.84	14.72
ROE_4	5.16	1.22	−0.60	0.22	0.638	0.872	0.41	12.68

**Table 3:** Fit indices for confirmatory factor analysis models.

Model	SSχ^2^	df	SSχ^2^/df	RMSEA [90% CI]	CFI	TLI	SRMR	WRMR
Oblique	300.050	98	3.062	0.065 [0.057; 0.073]	0.982	0.978	0.047	1.021
Higher-order	260.479	100	2.605	0.057 [0.049; 0.066]	0.986	0.983	0.049	1.047
Unifactorial	3091.281	104	29.724	0.243 [0.235; 0.250]	0.731	0.689	0.182	4.306
Bifactor	221.795	88	2.520	0.056 [0.047; 0.065]	0.988	0.984	0.043	0.925
Orthogonal	1365.138	104	13.126	0.158 [0.150; 0.165]	0.886	0.869	0.182	3.571

Note. SSχ^2^ = chi square (scale-shifted approach); df = degree of freedom; RMSEA = root mean square error of approximation; CI = confidence interval; CFI = comparative fit index; TLI = Tucker–Lewis index; SRMR = standardized root mean square residual; WRMR = weighted root mean square residual.

**Table 4:** Reliability, mean inter-item correlation, and convergent and discriminant validity evidence.

Variable	ω_cat_	Mean r_i-i_	AVE	SH	Discriminant Validity Evidence				
					SEA	OEA	UOE	ROE	SH
SEA	0.755	0.401	0.473	0.357	0.688 ^a^	0.421	0.592	0.473	0.473
OEA	0.801	0.509	0.537	0.007	0.368	0.733 ^a^	0.193	0.333	—
UOE	0.838	0.617	0.640	0.369	0.438	0.094	0.800 ^a^	0.250	0.414
ROE	0.914	0.746	0.764	0.362	0.440	0.217	0.157	0.874 ^a^	0.416
SH	0.763	0.572	0.594	—	0.357	0.007	0.369	0.362	0.771 ^a^
EI	0.628	0.273	—	0.552	—	—	—	—	—

Note. SEA = self-emotion appraisal; OEA = others’ emotion appraisal; UOE = use of emotion; ROE = regulation of emotion; SH = subjective happiness; ω_cat_ = categorical omega; AVE = average variance extracted; r_i-i_ = inter-item correlation. ^a^ In the discriminant validity evidence section, on diagonal, square root of the AVE; intercorrelations between variables are presented below the diagonal; heterotrait–monotrait ratio of correlations (HTMT2) are presented above the diagonal.

**Table 5:** Inter-item polychoric correlation matrix.

Item	SEA_1	SEA_2	SEA_3	SEA_4	OEA_1	OEA_2	OEA_3	OEA_4	UOE_1	UOE_2	UOE_3	UOE_4	ROE_1	ROE_2	ROE_3	ROE_4
SEA_1	—															
SEA_2	0.58	—														
SEA_3	0.54	0.65	—													
SEA_4	0.15	0.21	0.28	—												
OEA_1	0.14	0.23	0.24	0.24	—											
OEA_2	0.20	0.25	0.25	0.21	0.68	—										
OEA_3	0.12	0.14	0.22	0.17	0.30	0.43	—									
OEA_4	0.16	0.25	0.35	0.18	0.46	0.60	0.58	—								
UOE_1	0.25	0.20	0.18	0.17	0.13	0.16	0.08	0.17	—							
UOE_2	0.23	0.24	0.28	0.24	0.11	0.08	-0.01	0.12	0.51	—						
UOE_3	0.32	0.31	0.32	0.25	0.09	0.14	0.05	0.18	0.52	0.69	—					
UOE_4	0.32	0.34	0.28	0.28	0.10	0.15	0.02	0.15	0.50	0.68	0.81	—				
ROE_1	0.23	0.36	0.30	0.14	0.19	0.15	0.13	0.22	0.10	0.21	0.22	0.21	—			
ROE_2	0.23	0.41	0.27	0.10	0.15	0.15	0.12	0.22	0.14	0.21	0.21	0.21	0.85	—		
ROE_3	0.21	0.32	0.21	0.09	0.11	0.09	0.11	0.18	0.12	0.19	0.26	0.23	0.64	0.63	—	
ROE_4	0.28	0.40	0.33	0.16	0.16	0.19	0.09	0.25	0.20	0.23	0.29	0.25	0.78	0.82	0.75	—

## Data Availability

The data presented in this study are available on request from the corresponding author.
